# ATAK complex (adrenaline, takotsubo, anaphylaxis, and kounis hypersensitivity-associated coronary syndrome) related to latamoxef administration—a case report

**DOI:** 10.3389/fcvm.2024.1383903

**Published:** 2024-07-22

**Authors:** Sheng Li, Peng Ding, Chunxia Wang, Kunlan Long, Peiyang Gao

**Affiliations:** Department of Critical Care Medicine, Hospital of Chengdu University of Traditional Chinese Medicine, Chengdu, China

**Keywords:** Kounis syndrome, stress cardiomyopathy, adrenaline, allergy, latamoxef, case

## Abstract

**Background:**

Adrenaline, stress cardiomyopathy, allergic reactions, and Kounis syndrome (Adrenaline, Takotsubo, Anaphylaxis, Kounis Complex, ATAK) constitute a complex clinical syndrome often associated with endogenous or exogenous adrenaline. Due to its rapid onset, severity, and treatment challenges, it warrants significant attention from clinicians. This article reports a case of Type II Kounis syndrome combined with stress cardiomyopathy (ATAK) triggered by a latamoxef-induced allergy.

**Case report:**

A 67-year-old male patient with an acute exacerbation of chronic obstructive pulmonary disease was admitted to the respiratory department for treatment. The day before discharge, after receiving a latamoxef infusion for 27 min, the patient developed wheezing, dyspnea, chills, profuse sweating, and an elevated body temperature, necessitating transfer to the ICU for monitoring and treatment. The ECG suggested a suspected myocardial infarction, while bedside echocardiography showed a left ventricular ejection fraction of 40%, segmental dysfunction of the left ventricle, and apical rounding. Emergency coronary angiography revealed 50% segmental eccentric stenosis in the mid-segment of the left anterior descending branch and right coronary artery. The final diagnosis was Type II Kounis Syndrome combined with stress cardiomyopathy due to a latamoxef-induced allergy, i.e., ATAK. Despite aggressive treatment, the patient succumbed to severe cardiogenic shock on the third day in the ICU.

**Conclusion:**

ATAK is a critical condition that progresses rapidly. For patients experiencing severe allergic reactions, monitoring biomarkers such as Troponin and ECG changes is crucial for timely recognition. If a patient is diagnosed with Kounis syndrome, caution should be exercised in using adrenaline to prevent ATAK.

## Introduction

Kounis syndrome is a a coronary artery syndrome caused by the interaction between mast cells and inflammatory cells (1). In recent years there has been a significant increase in reported cases of Kounis syndrome, leading to increased understanding, and therefore the diagnosis rate of this disease has gradually increased. However, relevant guidelines are still lacking (2). Due to its association with stress cardiomyopathy, a new approach to combining the two has been proposed in recent years: Adrenaline, Takotsubo, Anaphylaxis, Kounis Complex (ATAK) (3). Although a very small number of case reports have demonstrated the challenging and complex nature of this disease complex in clinical practice, the academic community's understanding of it remains incomplete (4, 5).

Current research suggests that the occurrence of ATAK is related to endogenous and/or exogenous adrenaline (6). Allergic reactions induce mast cells and various inflammatory cells to cause Kounis syndrome, while the stimulation of inflammatory factors leads to the release of endogenous catecholamines. The use of exogenous adrenaline also increases the body's catecholamine levels, collectively triggering stress cardiomyopathy. The combined effects of these factors severely damage cardiac function, further deteriorating hemodynamics, and potentially leading to cardiac collapse (7). However, the exact pathogenesis of this disease complex is not yet fully understood. This article reports a case of ATAK possibly triggered by latamoxef infusion, but further research is needed to confirm the exact cause(s).

## Case report

**Figure 1:**
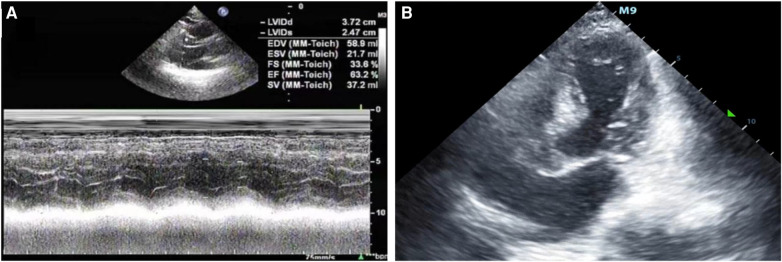
(**A**) The patient was admitted to the hospital with a left ventricular ejection fraction of 63.2% and normal systolic and diastolic function. (**B**) After admission to the ICU, bedside color ultrasound examination of the heart showed that the apex of the heart was round and blunt, with left ventricular systolic dysfunction and basal ganglia contraction.

**Figure 2:**
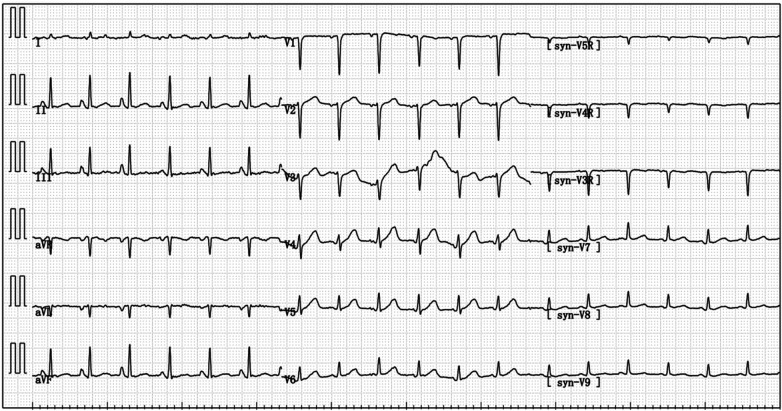
On admission, the electrocardiogram indicated sinus tachycardia; short PR interval; RV1–V3 increases poorly.

A 67-year-old male patient was admitted to our respiratory department on January 8, 2024, with the chief complaint of a “recurrent cough with sputum for over six years, aggravated for more than one month”. The admission echocardiogram (Figure 1A) and electrocardiogram (Figure 2) showed no significant abnormalities in cardiac structure and function. High-sensitivity troponin I was measured at 0.012 ng/ml (normal range: 0–0.0535 ng/ml), and BNP was 187.1 pg/ml (normal range: 0–125 pg/ml). The patient had a history of chronic obstructive pulmonary disease for over six years, no history of allergies, and no other significant medical history.

After admission, the patient received latamoxef for infection, theophylline for bronchodilation, and omeprazole for gastric acid suppression. After about a week of treatment, the patient's cough and sputum symptoms significantly improved, all indicators returned to normal, and no fever, chest tightness, wheezing, or any other discomfort remained. It was planned to discharge the patient on January 18. On January 8, the patient received intravenous latamoxef infusion (1.5 g, twice daily), and after each infusion he experienced palpitations and uneasiness, but the patient confirmed these symptoms were mild and tolerable. Therefore it did not raise concerns for the patient or the respiratory department physicians.

However, on the afternoon of January 17 at 15:40, approximately 27 mins after the last dose of latamoxef infusion, the patient developed dyspnea, difficulty breathing, chills, sweating, and fever. The latamoxef infusion was immediately stopped, and methylprednisolone 40 mg was administered intravenously. However, the patient's consciousness deteriorated further, with delayed responses, cold extremities, decreased blood pressure, pulse oximetry saturation, and respiratory status. The respiratory department urgently contacted the ICU for endotracheal intubation and mechanical ventilation assistance. The patient was transferred to the ICU for monitoring and treatment.

**Figure 3:**
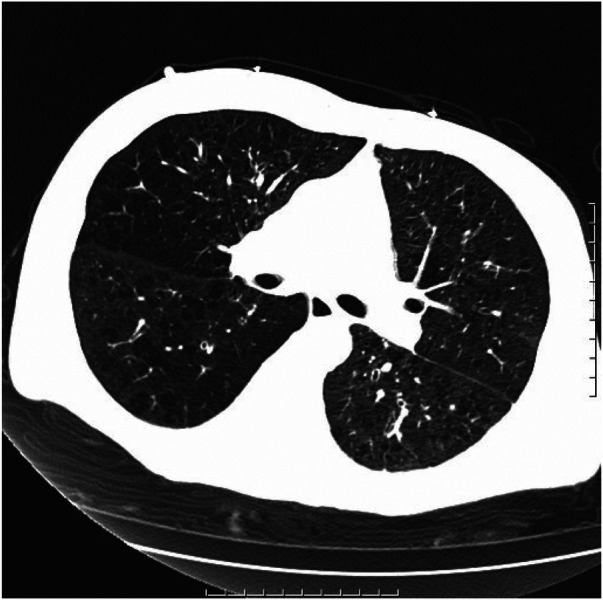
Chronic bronchitis and emphysema with multiple bulla formation; both lungs scattered in fibrous foci.

**Figure 4:**
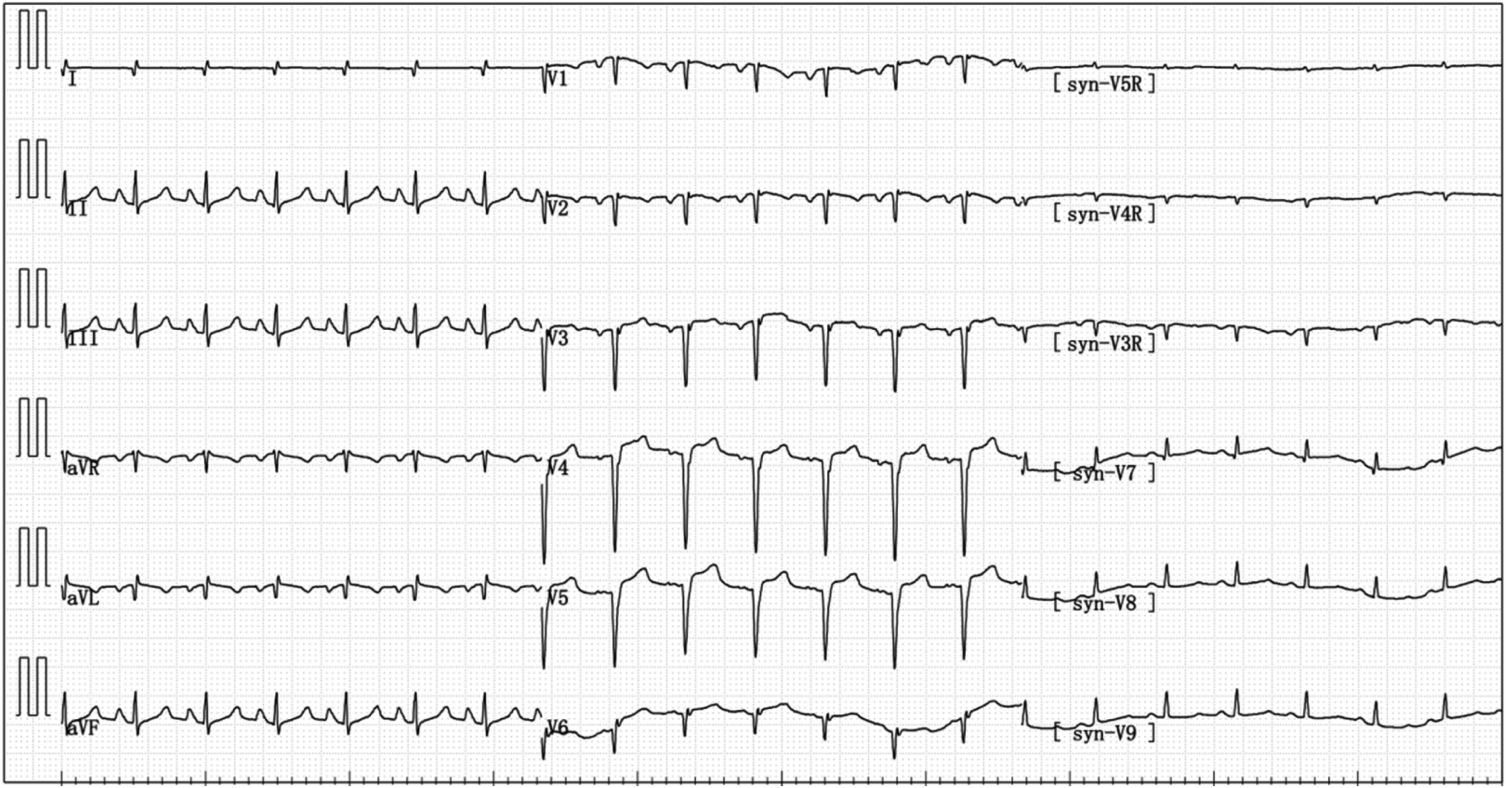
Electrocardiogram reexamination after the patient was transferred to ICU: abnormal q wave appeared in I and avL, and the ST segment of V3–V6 leads was raised by 0.05–0.15 mV.

After being admitted to the ICU, the patient quickly developed purpura on both lower limbs, causing the whole body feeling cold and clammy. At this time, the patient's vital signs were as follows: temperature of 36 degrees Celsius, heart rate of 152 beats per minute, blood pressure of 75/46 mmHg, respiratory rate of 35 breaths per minute, and oxygen saturation of 96%. We immediately administered epinephrine to maintain blood pressure, along with milrinone for cardiac support, and used a ventilator to assist the patient's breathing. Bedside echocardiography indicated the patient's left ventricular ejection fraction (EF) was 30%–40%, with segmental dysfunction of the left ventricle and a rounded apex, suggesting the possibility of stress-induced cardiomyopathy (Figure 1B). Chest CT indicated bronchospasm without clear signs of infection (Figure 3). Emergency blood tests showed high-sensitivity troponin I at 3.24 ng/ml and BNP at 4850.2 pg/ml. The electrocardiogram showed extreme clockwise rotation, left atrial abnormality, abnormal Q waves in leads I and aVL, poor progression of R waves from leads V1 to V6, ST segment elevation of 0.05–0.15 mV in leads V3–V6, and T-wave changes (Figure 4). After a consultation with the cardiology team, emergency percutaneous coronary intervention (PCI) was recommended.

**Figure 5:**
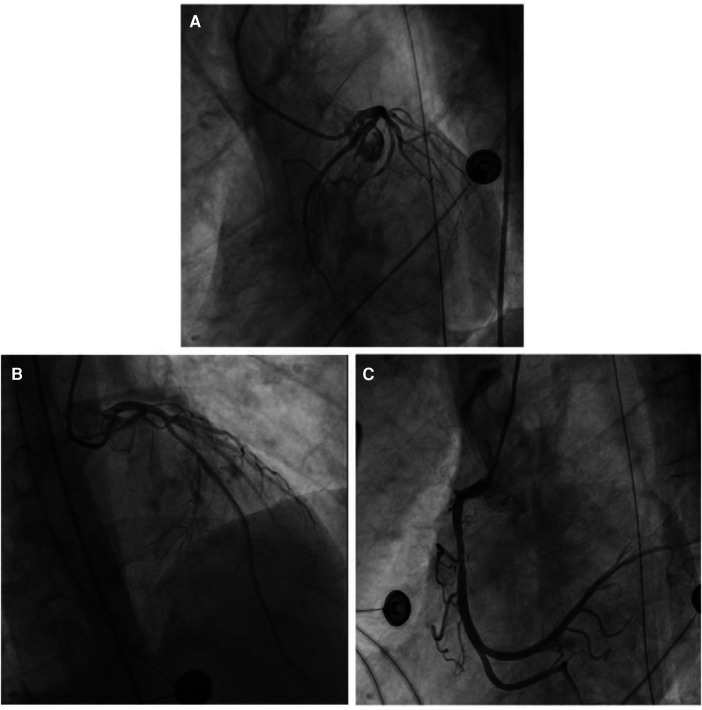
(**A**) No obvious abnormalities were noted in the left circumflex coronary artery. (**B**) The most severe segmental eccentric stenosis of about 50% was observed in the middle segment of the left anterior descending coronary artery. (**C**) The most severe segmental eccentric stenosis of about 50% was observed in the middle segment of the right coronary artery.

Emergency coronary angiography revealed approximately 50% eccentric stenosis in the mid-segment of the left anterior descending coronary artery and approximately 50% eccentric stenosis in the mid-segment of the right coronary artery, with no significant abnormalities in other vessels (Figure 5). Following the coronary angiography procedure, the patient returned to the ICU for further treatment. Hemodynamic monitoring showed: cardiac output (CO) of 3.4 L/min, cardiac index (CI) of 1.9 L/min/m^2^, stroke volume (SV) of 27 ml, stroke volume index (SVI) of 16 ml/m^2^, systemic vascular resistance (SVR) of 1,250 dynes·s/cm^5^, systemic vascular resistance index (SVRI) of 2,192 dynes·s/cm^5^/m^2^, extravascular lung water (EVLW) of 435 ml, extravascular lung water index (EVLWI) of 6.6 ml/kg, global end-diastolic volume (GEDV) of 828 ml, and global end-diastolic volume index (GEDVI) of 469 ml/m^2^, consistent with features of cardiogenic shock.

Although the patient's blood tests did not show a significant increase in eosinophils and blood IgE levels, the evidence for diagnosing allergic diseases seemed insufficient, which is why anti-allergy treatment was not initially administered. However, results from tests such as chest CT, head CT, blood cultures, abdominal ultrasound, and coronary angiography ruled out conditions like sepsis-induced septic myocarditis, acute myocardial infarction, or structural heart diseases. Considering the patient's medical history and presentation at the onset of symptoms, it is still plausible to consider coronary artery spasm due to a latamoxef allergy leading to Kounis syndrome, possibly compounded by stress-induced cardiomyopathy. Therefore, the patient was continuously given norepinephrine to maintain blood pressure, milrinone for cardiac support, and appropriate fluid replacement to optimize preload. Despite discontinuing the use of epinephrine, the patient's condition worsened, and the family decided to withdraw life-saving treatments. The patient passed away on January 19, 2024. Cause of death: cardiogenic shock.

## Discussion

The concept of Kounis syndrome was first proposed in 1991 (8), and to date there have been over 400 related case reports, with a growing amount published in recent years. It is currently believed that the occurrence of Kounis syndrome is associated with mast cell-mediated allergic reactions, where an allergic reaction induces coronary artery spasm with or without rupture of atherosclerotic plaques. Based on the severity of coronary artery involvement, Kounis syndrome can be classified into three types: Type I involves essentially normal coronary arteries, with myocardial ischemia due to coronary spasm, possibly with or without elevation of cardiac injury markers; Type II occurs in patients with pre-existing atherosclerosis, where an allergic reaction triggers coronary spasm or rupture of atherosclerotic plaques leading to further narrowing of the vessel lumen; Type III occurs in patients with a history of coronary artery stent implantation, where an allergic reaction leads to stent thrombosis (9). Recently, Giovannini et al. proposed a fourth type of Kounis syndrome, suggesting that an allergic reaction can lead to thrombosis post-coronary artery bypass graft surgery (10).

Food, drugs, and environmental exposure can all potentially lead to the occurrence of this syndrome. Antibiotics are one of the significant causes of Kounis syndrome, with approximately 80% of reported cases experiencing symptoms within 30 min of drug exposure (11). However in some cases, similar to the one reported in this study, patients do not exhibit severe allergic reactions immediately upon initial drug exposure, making diagnosis more challenging (12). Diagnosing Kounis syndrome poses a certain challenge in clinical practice. Some biomarkers are meaningful for diagnosis, such as serum tryptase, histamine, cardiac enzyme profile, cardiac troponin, and pancreatic enzymes. If a patient experiences palpitations, chest pain, or other discomfort symptoms after an allergic reaction, electrocardiography, echocardiography, and coronary angiography are also crucial for diagnosis. Even in severe allergic reactions, IgE levels can still be negative (13). In non-allergic acute myocardial infarction and unstable angina patients, pancreatic enzymes may still be significantly elevated (14), possibly due to the presence of chronic inflammation in atherosclerotic plaques (15). Therefore, the diagnostic approach for Kounis syndrome is limited, with a special history of drug exposure, occurrence of allergic reactions, and corresponding symptoms of coronary artery involvement being the main basis for diagnosis (16). At the same time, it is worth noting that the occurrence of Kounis syndrome may not be associated with common allergic symptoms, such as skin rashes, itching, etc (16, 17), making the diagnosis of Kounis syndrome challenging.

Stress-induced cardiomyopathy (TTS) was first named by Sato et al. due to its typical and unique cardiac manifestations that differentiate it from other cardiac conditions, such as left ventricular apical dysfunction, apical ballooning, basal hyperkinesis, and non-obstructive coronary arteries (18). The exact pathogenesis of stress-induced cardiomyopathy is not fully understood, but excessive release of catecholamines is considered a key factor, leading to direct catecholamine toxicity, damage mediated by adrenergic receptors, pericardial and microvascular coronary artery vasoconstriction and/or spasm, and increased cardiac workload (19). TTS and acute coronary syndrome (ACS) are very similar in clinical presentation and ST-segment elevation, making it difficult to distinguish them in the early stages of the disease. Studies have shown that the incidence of TTS accounts for about 2%–3% of suspected ACS patients (20), and echocardiography and coronary angiography play important diagnostic roles during this period (19).

**Figure 6:**
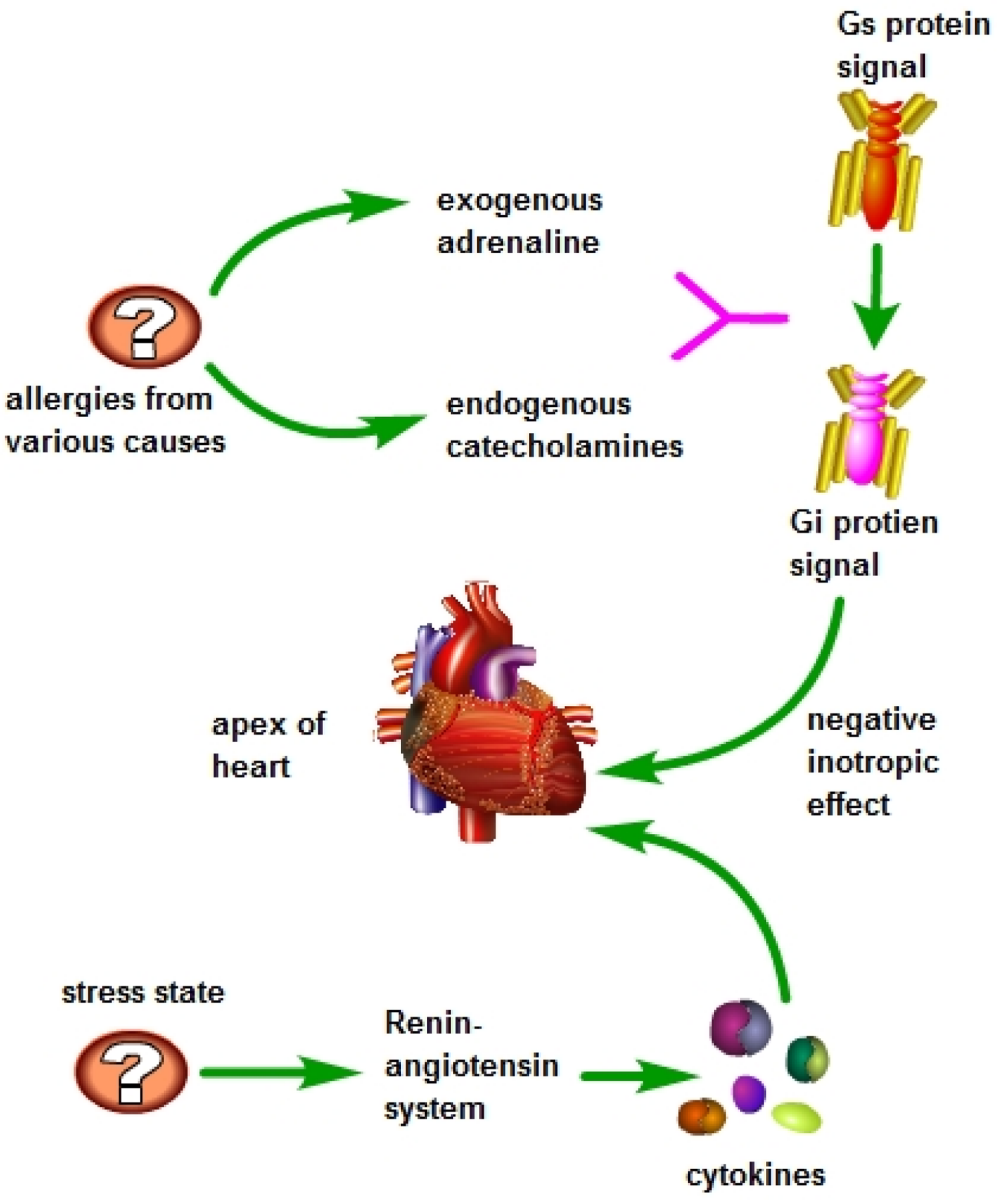
During allergic reactions, the body releases significant amounts of catecholamines, and during the treatment process, exogenous epinephrine is administered. High doses of epinephrine are known to trigger a shift in signal transmission within ventricular myocytes, specifically converting the Gs protein signal to a Gi protein signal through the β2-adrenergic receptor. This conversion results in a notable negative inotropic effect, particularly pronounced at the apex of the heart. Additionally, during periods of stress, activation of the sympathetic nervous system stimulates the renin-angiotensin system, leading to the production of cytokines and activation of mast cells and other key mechanisms. This cascade of events can potentially induce Kounis syndrome in a reverse manner.

In 2006, a case report of a female patient with TTS caught the attention of Professor Kounis, who believed that there might be some connection between TTS and Kounis syndrome, but further exploration was needed (21). In 2009, Japanese scholars first reported a case that was clearly related to Kounis syndrome and TTS (22). In 2016, Professor Kounis officially introduced the concept of ATAK, suggesting that its pathogenesis is similar to stress-induced cardiomyopathy caused by pheochromocytoma, with excessive release of catecholamines as the basis of the disease (23). It is currently believed that during allergic reactions, the body releases a large amount of catecholamines, and the use of exogenous adrenaline during treatment triggers the conversion of intracellular signal transduction in ventricular myocytes, specifically through the β2-adrenergic receptor, switching from Gs protein signaling to Gi protein signaling, resulting in a certain negative inotropic effect, which is more pronounced at the apex of the heart (24, 25). Additionally, under stress conditions, sympathetic nervous system stimulation activates the renin-angiotensin system, leading to the production of cytokines and the activation of core mechanisms such as mast cells, which can also trigger Kounis syndrome in a reverse manner (Figure 6) (6).

Currently, there is limited research on ATAK in epidemiology. A study conducted in the United States showed that among patients with Kounis syndrome combined with TTS, the majority were white women, and this population had more cardiovascular risk factors and higher resource utilization rates (26). There is no related research on the incidence of ATAK at present. After the concept of ATAK was proposed, 11 articles explicitly reported related cases supporting this hypothesis. Although the number of cases seems small, more articles have pointed out the risks of using adrenaline in the treatment of allergic diseases, which may lead to cardiovascular events (27, 28). This indirectly indicates that the concept of ATAK has not been widely discussed, but it holds certain importance in clinical practice.

For the diagnosis of ATAK, similar to Kounis syndrome and TTS, myocardial injury markers, BNP, D-dimer, complete blood cell count, amylase, histamine, electrocardiogram, echocardiography, and coronary angiography are important (7). For patients with an unclear diagnosis, it is necessary to complete a thorough inquiry into medical history.

Latamoxef is a semi-synthetic cephalosporin antibiotic drug, whose antibacterial mechanism mainly involves binding to target proteins on the cell membrane to prevent bacteria from dividing normally (29). There have been no reports of II-type Kounis syndrome combined with stress-induced cardiomyopathy caused by latamoxef. In the case reported in this article, the patient developed obvious shortness of breath, chest tightness, widespread wheezing in both lungs, and then rapidly developed symptoms such as hypotension after receiving latamoxef infusion. The patient's blood eosinophil and IgE were negative in the initial screening, so the possibility of Kounis syndrome was not considered at the beginning of the treatment. The patient's coronary angiography was negative, and after being admitted to the ICU, chest CT, abdominal ultrasound, blood culture, and other tests were performed to rule out the possibility of infectious diseases. We then carefully inquired about the patient's medical history and changes in symptoms during the onset, which were consistent with bronchospasm caused by allergies. Therefore, this is considered to be a case of II-type Kounis syndrome caused by latamoxef infusion.

Upon admission to the ICU, the patient's echocardiogram showed a decreased ejection fraction and a rounded apex, ruling out the possibility of structural heart disease. Elevated levels of cardiac enzymes and BNP and hemodynamic monitoring indicators were observed, consistent with stress-induced cardiomyopathy complicated by severe cardiogenic shock. Additionally, the diagnosis of aortic dissection was ruled out due to the absence of obvious tearing chest pain in the patient and minimal difference in blood pressure in the limbs. Pulmonary embolism was also excluded as the patient's D-dimer levels did not show a significant increase. The chest CT scan did not reveal signs of pulmonary edema, and auscultation of the lungs did not indicate significant crepitations, thereby excluding the possibility of acute heart failure.

It is noteworthy that the severe symptoms in this patient only appeared on the 10th day after the administration of ceftriaxone, which differs from most existing studies on Kounis syndrome. Drug hypersensitivity reaction syndrome may explain this rare occurrence, which is a delayed allergic reaction mediated by T cells (30). Although the pathogenesis is not clear, retrospective case studies have shown that the incidence of myocardial damage caused by drug hypersensitivity reactions is approximately between 4% and 21% (31–33). This damage can occur either in the early stage (within one week) or later than 5 weeks after the onset of the disease (34). Therefore, we still consider this patient to have had ATAK. Unfortunately, the patient's condition was critical, and a cardiac MRI was not promptly conducted to definitively diagnose stress-induced cardiomyopathy.

Following this case we recognize that ATAK is an exclusive diagnosis. This means that when patients have chest pain, difficulty breathing and other symptoms, medical professionals must consider whether patients have acute myocardial infarction, pulmonary embolism, aortic dissection, acute heart failure or acute myocarditis. It is necessary to conduct auxiliary tests according to the corresponding symptoms and signs, such as cardiase, hypersensitive troponin, BNP, electrocardiogram, coronary angiography, echocardiography, etc. We believe, because they are more readily available than cardiac MRI, coronary angiography and echocardiography play an important role in this process. Although the patient did not exhibit some common allergic symptoms, such as skin redness and itching, we cannot completely rule out the possibility of an allergic reaction. Obtaining a patient's medical history is crucial to the diagnosis of ATAK, as it informs the likelihood of an allergic reaction. We also believe that it is necessary to test for biomarkers of allergic reactions, but a negative result does not completely rule out the possibility of allergy.

Currently, there is no systematic treatment plan for ATAK. Antihistamines, antiplatelet agents, relieving vascular spasm, and promptly opening the occluded vessels are important methods for treating Kounis syndrome (35). Adrenaline remains the first-line medication for treating allergic reactions. According to international guidelines, the recommended route of administration for adrenaline is intramuscular injection, with a maximum adult dose of 0.5 mg. Second-line medications include antihistamines and corticosteroids. Antihistamines may cause hypotension in patients, and are not suitable for those with severe shock (30). Short-acting corticosteroids are effective and relatively safe. For patients with concomitant TTS, drug therapy is not the primary approach. Mild patients may not require treatment or only limited short-term treatment, while severe patients should consider early use of mechanical support as a bridge to recovery. The main goal of in-hospital treatment should be supportive care, life maintenance, and complication reduction.

This report has certain limitations. Firstly, the experiences presented through the diagnosis and treatment process of this patient may not be generalizable. Secondly, the absence of clinical information due to the patient's family opting to discontinue treatment limited our ability to conduct pancreatic enzyme, histamine, and serum tryptophan testing, as well as early initiation of mechanical support, which may have impacted the patient's prognosis. However, a key strength of this report is that it has reported the first case of II-type Kounis syndrome combined with stress-induced cardiomyopathy caused by latamoxef.

## Conclusion

ATAK is a clinically challenging syndrome that is difficult to diagnose and treat, deserving the attention of clinicians. Endogenous and exogenous adrenaline play an important role in its pathogenesis. For patients diagnosed with Kounis syndrome or clear allergies, it is important to monitor changes in the patient's cardiac function, electrocardiogram, and myocardial injury markers. Careful consideration of the benefits and potential harms of adrenaline use is essential.

## Data Availability

The original contributions presented in the study are included in the article/Supplementary Material, further inquiries can be directed to the corresponding author.
